# Assessment of Modality and Accuracy of Single Root Canal Treatment Performed by Undergraduate Students in Saudi Arabia: A Retrospective Study

**DOI:** 10.7759/cureus.33483

**Published:** 2023-01-07

**Authors:** Turki A Alshehri, Abdullah Aljami, Haneen Alzayer, Hussain Aljubran, Muhammad A Faridi, Soban Q Khan, Abdul Khabeer

**Affiliations:** 1 College of Dentistry, Imam Abdulrahman Bin Faisal University, Dammam, SAU; 2 Department of Restorative Dental Sciences, College of Dentistry, Imam Abdulrahman Bin Faisal University, Dammam, SAU; 3 Department of Dental Education, College of Dentistry, Imam Abdulrahman Bin Faisal University, Dammam, SAU; 4 Undergraduate Medical Education, Schulich School of Medicine and Dentistry at University of Western Ontario, London, CAN

**Keywords:** perforation, ledge, procedural errors, endodontic mishap, root canal treated teeth, root canal obturation, root canal therapy

## Abstract

Aim

To assess the radiographic quality of root canal treatment (RCT) performed on single-rooted anterior teeth by undergraduate dental students of Imam Abdulrahman Bin Faisal University (IAU). Moreover, the study also aimed to assess the types of procedural errors encountered during root canal treatment and to compare the results between male and female students.

Methodology

The record of patients who visited the endodontic clinics at IAU between the years 2018 and 2021 was obtained from the medical records department. The inclusion criteria for the study involved: i) RCT performed on anterior teeth with a single root; ii) RCT performed by fourth, fifth, and sixth-year undergraduate dental students; and iii) availability of pre-operative and post-operative peri-apical radiographs. After the inclusion criteria, a total of 278 records were selected. The radiographs were accessed by two calibrated examiners for the length of the obturation, homogeneity, and taper. In addition, procedural errors such as the presence of a ledge, perforation, or fractured instruments were also observed. Analysis was done using the Chi-square test.

Results

A total of 139 teeth (50%) were found to have an adequate quality root canal treatment. Regarding the length of the obturation, 85.6% were considered acceptable, while 65.1% of the obturations had acceptable radiographic homogeneity. The acceptable taper was found in 71.9% of the obturations. Dental students who participated in this study demonstrated a low rate of procedural errors, with 4.7% ledge formation and 1% perforation. A statistically significant difference was found in the quality of root canal obturation (P *= *<0.001) performed by fourth, fifth, and sixth-year students. Moreover, a significant difference was also observed between maxillary and mandibular teeth (P= 0.032).

Conclusion

The quality of RCT performed by undergraduate dental students demonstrated that improvements are required. The teaching methods used in the endodontic courses need to be developed and improved to ensure the best possible learning and treatment outcomes.

## Introduction

The primary aim of root canal treatment (RCT) is to eliminate the microorganisms and their toxins that lead to acute and chronic inflammatory responses [1]. Endodontics is one of the most difficult subjects in the dental curriculum as it demands technical precision depending on the case complexity [2]. Undergraduate dental students attain theoretical and practical knowledge of endodontics during pre-clinical and clinical endodontic courses, which helps them diagnose and manage clinical practice accurately [1,3]. To pass the pre-clinical and clinical endodontic courses, the students are usually required to complete a certain number of cases and successfully appear for the competency examination. This ensures that the students have attained experience in performing RCT, which allows them to gain confidence and their judgmental capabilities [2]. The American Association of Endodontics, which aids in executing standardized clinical root canal treatment [4,5], has described endodontic guidelines. This guideline ensures that optimal treatment should be available for all patients. Treatment quality can be assessed radiographically according to specific parameters that include length, homogeneity, and taper of obturation, while clinically relieving signs and symptoms, diminished swelling, and absence of sinus tracts if present [6].

Several studies have investigated the performance of RCT by undergraduate dental students. A study conducted by Elemam RF et al., 2015 at Libyan International Medical University reported that the length of obturation in canines was better compared to central incisors. In contrast, the lower premolars had the most inadequate obturation length. In addition, the homogeneity of root canal obturation in the lower premolars was 100% adequate, while the maxillary canines showed a similar taper to mandibular premolars [7]. Another study conducted by Eskandarloo A et al., 2017 at Hamadan Dental School in Iran reported that the length of obturation in incisors was better compared to premolars and molars. The homogeneity of obturation in maxillary and mandibular premolars was adequate in 34% of root canal-treated teeth, while the highest quality of tapering was noted in premolars [5]. A study was done at Queen’s University, Belfast, United Kingdom, reported that 39% of anterior teeth had inadequate obturation [8]. The radiographic evaluation of RCT was conducted by the University of Espírito Santo (UFES), Brazil [9], and a study conducted at Suleyman Demirel University, Turkey showed that the quality of obturation performed by first and second-clinical-year students was almost similar [6]. Lastly, a study conducted at Taibah University in Saudi Arabia assessed the average technical quality of obturation performed by the fourth and fifth years. They reported no significant difference between fourth and fifth-year students. However, the fifth-year students showed more procedural errors, specifically inadequate obturation length, compared to fourth-year students, which they attributed to the high number of cases performed by fifth-year students [10]. Undergraduate dental students also assessed the quality of RCT from Jordan, Greece, and Saudi Arabia [11-13]. Balto H et al., 2010 conducted a study at a dental college in Riyadh, Saudi Arabia, and reported that there was no significant difference in the obturation length and taper performed by fourth and fifth-year students [14].

Currently, there is a lack of research investigating the quality of RCT performed by undergraduate dental students in the Eastern Province of Saudi Arabia. Comparing the quality of root canal treatment among different year levels can provide insight into the effect of the pre-clinical and clinical endodontic courses on the quality of the RCT procedure performed and can help to improve the established curriculum. Also, considering the fact that male and female students get their training in separate sessions may play a role in delivering different knowledge or experiencing different practices. Therefore, this study aims to assess the radiographic quality of root canal treatment performed on single-rooted anterior teeth by an undergraduate dental student of IAU, Saudi Arabia. This study also aims to assess the procedural errors encountered during root canal treatment and to compare the results between male and female students.

## Materials and methods

A retrospective cross-sectional study model was conducted to evaluate the current study outcome. Ethical approval (IRB-2022-02-367) was obtained from the Institutional Review Board (IRB) at IAU. The patients who visited the dental hospital clinics between 2018 and 2021 and underwent an RCT were included. A simple random sampling of 450 RCT cases was obtained from the records of the dental hospital at IAU. A sample size of 278 was obtained by determining the RCT procedures performed on anterior teeth with a single canal by 4th, 5th, and 6th-year undergraduate dental students between May 2018 and May 2021. The included records were the patient's age, gender, and procedure performed besides periapical radiographs (PA). Inclusion criteria included all records containing a detailed treatment report of the type of local anesthesia used, rubber dam isolation, working length measurements, step-back cleaning and shaping technique using K-files (Dentsply Maillefer, Ballaigues, Switzerland), and irrigation using 0.5% sodium hypochlorite. All teeth were obturated using 2% taper gutta-percha points (Sure Dent Corporation, Gyeonggi-do, South Korea) and AH plus sealer (Dentsply, DeTrey, Konstanz, Germany) using the lateral condensation technique. Pre-operative and post-operative PA radiographs taken by digital sensors (Gendex GXS-700, DEXIS, Sydney, New South Wales) were mandatory for evaluation. The PA radiographs were taken using a parallel technique with the help of an endodontic procedure-specific positioner. The exposure time varied between 0.08 and 0.13 seconds, while a voltage of 70 kV was used. All records with poor-quality radiographs, missing treatment details, and cases with patients younger than 18 years of age were excluded from the current study.

The digital PA radiographs were observed on an LED computer screen using the magnification and measurement tool available in the radiographic software (MiPACS Dental Enterprise Viewer 3.1.1404, Medicor Imaging, Charlotte, NC) at the college of dentistry [11]. Two endodontists with a master's degree and more than 10 years of clinical experience evaluated the PA radiographs. Inter-examiner reliability was calculated to evaluate the degree of agreement between two examiners by examining 15 single-canal RCT cases. Kappa statistics resulted in a value of 0.865, showing a high level of agreement between the examiners. Evaluation of radiographs was made in accordance with the criteria reported by Barrieshi-Nusair KM et al. and Matoug-Elwerfelli M et al. (Table 1) [11,15].

**Table 1:** The criteria to record information from radiographs.

Variable	Criteria	Definition
Length of root canal filling	Acceptable	Root filling ending < 2mm short of the radiographic apex.
	Over	Root filling ending beyond the radiographic apex.
	Under	Root filling ending > 2mm short of the radiographic apex.
Homogeneity of root canal filling	Acceptable	Uniform homogeneity of root filling without voids and canal space is not visible.
	Poor	Not uniform homogeneity of root filling with a clear presence of voids and canal space is visible.
Taper of root canal filling	Acceptable	Consistent taper from the coronal to the apical part of the filling, with a good, reflected canal shape.
	Poor	No consistent taper from the coronal to the apical part of the filling.

The overall quality was considered adequate only if the obturation length, homogeneity, and taper were all acceptable. In addition, procedural errors were evaluated according to the criteria described by Khabbaz MG et al. and Al-Khafaji T et al. (Table 2) [12,16]. 

**Table 2:** Criteria for the detection of procedural errors.

Parameters	Description
Ledge	A ledge was identified if the root filling on the final radiograph did not follow the curvature of the main canal path on the working length radiograph.
Root perforation	Root perforations (including furcation perforation, strip perforation, and lateral perforations of the root) were detected when extrusion of the filling materials was identified in any area of the root except the apical foramen.
Foramen perforation	Foramen perforation was diagnosed when the apical termination of the filled canal appeared as an elliptical shape transported to the outer wall.
Fractured instrument	Fractured instrument was detected by observing a part of the instrument in the root canal system or in the periradicular area on the final radiograph.

All collected data were entered on an Excel sheet and then exported for statistical analysis on IBM SPSS Statistics for Windows, Version 29.0 (IBM Corp. Armonk, New York). Average, SD, frequency distribution and graphs were calculated and prepared under descriptive statistics and Chi-square as part of interventional statistics.

## Results

A total of 278 anterior teeth were included as per the inclusion criteria. Of these teeth, 149 (53.6%) were treated by male students and 129 (46.4%) by female students. The number of teeth treated by different years of students and the distribution according to their location in the arch is mentioned in Table 3. Most of the teeth (47%) were treated by 5th-year students, and 80.9% of the total teeth were from the maxillary arch. 

**Table 3:** Distribution of teeth (N=278).

Variable	N (%)
Student gender	
Male	149 (53.6)
Female	129 (46.4)
Year level	
Fourth year	70 (25.2)
Fifth year	132 (47.5)
Sixth year	76 (27.3)
Arch	
Maxillary	225 (80.9)
Mandibular	53 (19.1)

The length, homogeneity, and taper of root canal obturations are shown in Table 4. The length of obturation was found acceptable in 238 (85.6%) teeth, while 181 (65.1%) of the obturations had acceptable radiographic homogeneity. The acceptable taper was observed in 200 (71.9%) teeth.

**Table 4:** Length, density and taper of root canal obturations.

Number of teeth	Length			Homogeneity		Taper	
278	Acceptable (%)	Over (%)	Under (%)	Acceptable (%)	Poor (%)	Acceptable (%)	Poor (%)
	238 (85.6)	20 (7.2)	20 (7.2)	181 (65.1)	97 (34.9)	200 (71.9)	78 (28.1)

**Figure 1:**
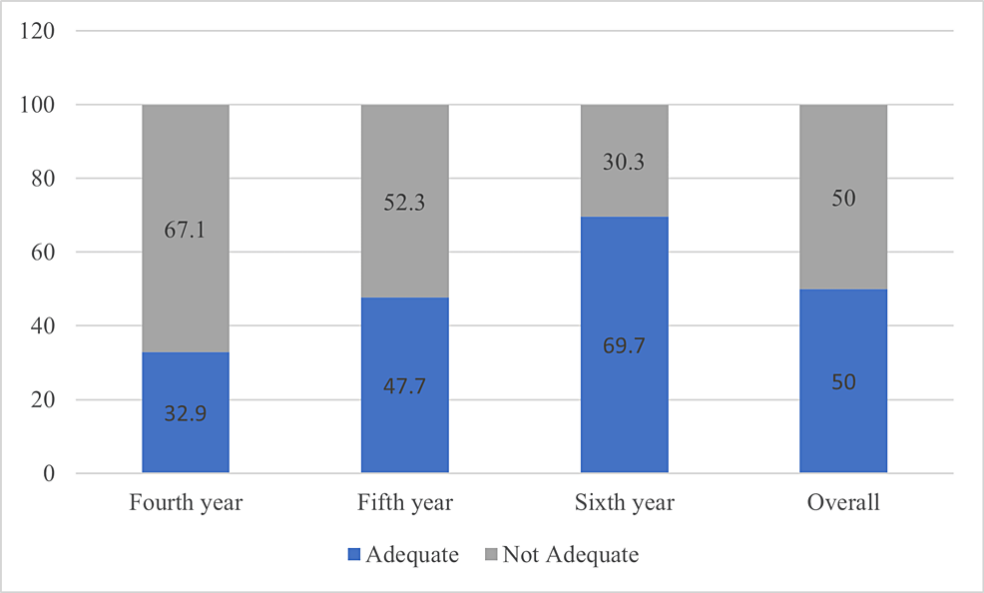
Quality of root canal obturation by different year level students.

The quality of root canal treatments performed by undergraduate students of different years is shown in Figure 1. A 32.9% of the root canal obturations performed by the fourth-year students were rated as adequate. Fifth-year students performed less than half (47.7%) of their treatments within the adequate criteria. Moreover, 69.7% of the root canal obturations performed by sixth-year students were adequate. This shows a steady increase in the student's skill level as they progress through their undergraduate years while performing root canal treatments. Overall, there was an equal (50%) distribution between the adequate and not adequate root canal treatments performed by all year levels

Cases evaluated in this study showed a low rate of procedural errors (Table 5). Only 4.7% of the assessed teeth showed a ledge formation. When assessing perforation, 99.6% of the assessed teeth were identified with no perforation. No fractured instruments were found among all the assessed teeth. No significant difference was found among the academic year in relation to the frequency of procedural errors determined.

**Table 5:** Frequency of procedural errors.

Mishaps	N (%)
Ledge	
Yes	13 (4.7)
No	265 (95.3)
Root perforation	
Yes	1 (0.4)
No	277 (99.6)
Foramen perforation	
Yes	2 (0.7)
No	276 (99.3)
Fractured Instrument	
Yes	0 (0)
No	278 (100)

A statistically significant difference was found between fourth, fifth, and sixth-year students according to the quality of root canal obturation (P= <0.001). A significant difference was also found between maxillary and mandibular teeth (P=0.032) (Table 6). No significant difference was found between male and female students' quality of root canal obturation and the frequency of procedural errors.

**Table 6:** Quality of root canal treatment and its relationship with study variables.

Variables	Adequate	Not Adequate	P-value
Gender			
Male	69 (46.3)	80 (53.7)	0.186
Female	70 (54.3)	59 (45.7)	
Year level			
Fourth year	23 (32.9)	47 (67.1)	<0.001*
Fifth year	63(47.7)	69 (52.3)	
Sixth year	53 (69.7)	23 (30.3)	
Arch			
Maxillary	120 (52.3)	34 (64.2)	0.032*
Mandibular	19 (35.8)	105 (46.7)	

## Discussion

The aim of this study was to assess the radiographic quality and procedural errors of root canal treatment performed by 4th, 5th, and 6th-year dental students at the college of dentistry at IAU. Treatment quality was assessed radiographically according to specific parameters, including obturation length, homogeneity, and taper. Procedural errors were assessed according to the presence of any ledge, perforation, or broken instrument.

The results of the current study showed that 50% of the root canal treatments performed by students were categorized as "adequate." This finding is consistent with another study published in Greece, which found that 55% of root canal fillings performed by dental students were acceptable [12]. However, in Saudi Arabia, the quality of root canal obturation performed by dental students was higher (63%) [17]. In Oklahoma, United States, 91% of root canal treatments were found to be successful and acceptable after radiographic evaluation [18].

Inadequate root canal treatments found in the current study can be attributed to the procedural errors performed by students. It has been reported that inexperienced dental students performing chemo-mechanical preparation with a step-back technique can lead to inadequate preparation of the root canal or further procedural errors such as ledges, strip-perforations, and apical transportation [17,18]. In addition, cold lateral condensation is a sensitive technique and can lead to voids and underfilled canals [17, 18].

Root canal treatments performed on anterior maxillary teeth were significantly greater quality when compared to mandibular anterior teeth (P=0.032). However, factors such as root anatomy, accessibility, the success of local anesthesia, and proximity to vital structures can increase difficulty while working on mandibular teeth [19].

The highest percentage of adequate root canal treatments was found among sixth-year students, followed by fifth-year students, and the least was found among fourth-year students. This can be explained by their experience in providing root canal treatments on even more difficult cases compared to fourth- and fifth-year students who have not yet treated as many patients.

The current study found a low frequency of procedural errors among anterior teeth performed by dental students. Only three perforations, 13 ledges, and no broken instruments were found during the radiographic evaluation. However, this finding might not be reliable because of the 2D radiographic assessment, which hinders the illustration of all procedural errors. Therefore, further Cone Beam Computed Tomography (CBCT) is required for more accurate detection and evaluation of root canal treatments [12]. In Greece, a higher prevalence of ledge (55%) root perforation (32%) and separated instruments (0.9%) was found in a 2D radiographic PA evaluation of mandibular and maxillary anterior and posterior teeth performed by fifth- and sixth-year students [12].

At the college of dentistry at IAU, the preclinical endodontic course has taken over two academic terms. Although thorough training according to pre-set learning objectives is followed during the preclinical curriculum, the short term can limit students' ability to improve their skills, such as canal preparation and practicing different obturation techniques. Another study in the United Kingdom has also expressed the need for more extended training periods for undergraduate dental students during the preclinical endodontic courses [20].

A limitation of this study could be that those anterior single-canal teeth tend to have an easier handling process than posterior multi-rooted teeth with multiple canals, which may not present the actual students' skills in performing adequate obturation forms. Also, it would help appraise students' self-efficacy in performing RCT procedures to ensure better technical management behavior. In addition, evaluating a 2D image does not provide an accurate interpretation of the obturation condition inside the canal. Also, the number of cases handled by 4th and 6th-year students is much less compared to 5th-year students, which may affect the presented percentages.

## Conclusions

Radiographic quality assessment of the root canal treatment performed by undergraduate dental students can help improve and develop the teaching strategies provided during the endodontic course to ensure optimum learning and treatment outcomes as it provides accurate specifications of the areas of strengths and weaknesses. This can be obtained by increasing the teacher-to-student ratio, allowing flexible training periods, and increasing preclinical and clinical requirements.
